# Nurses’ knowledge, attitude, and competence regarding palliative and end-of-life care: a path analysis

**DOI:** 10.7717/peerj.11864

**Published:** 2021-07-26

**Authors:** Hung-Yu Lin, Chun-I Chen, Chu-Yun Lu, Shu-Chuan Lin, Chiung-Yu Huang

**Affiliations:** 1 School of Medicine, College of Medicine, I-Shou University, Kaohsiung, Taiwan, ROC; 2 Division of Urology, Department of Surgery, E-Da Cancer & E-Da Hospital, Kaohsiung, Taiwan, ROC; 3 Management College, I-Shou University, Kaohsiung, Taiwan; 4 Department of Nursing, I-Shou University, Kaohsiung, Taiwan; 5 Department of Nursing, E-Da Hospital, Kaohsiung, Taiwan

**Keywords:** Attitude, Palliative knowledge, Self-competence, End-of-life

## Abstract

**Background:**

Nurses’ knowledge regarding palliative and end-of-life (EOL) care has been documented, but the competence of nurses in Taiwan has not been deeply analyzed and may affect the use of EOL care.

**Purpose:**

We aimed to (1) assess the palliative care knowledge, competence and attitude of nurses in a general hospital and (2) examine the paths connecting nurses’ demographic characteristics, previous experiences, knowledge, competence, and attitude.

**Method:**

A correlational, cross-sectional survey design was implemented to recruit 682 eligible nurses. The questionnaires included demographic information and palliative and EOL care knowledge, attitude, and competence scales. Path analysis was employed for statistical analysis using structural equation modeling.

**Results:**

Overall, 76% of the questions assessing palliative and hospice knowledge were answered correctly. Nurses’ palliative attitudes were divided into “positive perception” and “negative perception”. “Positive perception” was highly correlated with competence (*r* = 0.48, *p* < 0.001), but “negative perception” was not significantly correlated with competence (*r* = −0.07, *p* = 0.25). “Positive perception” (*β* =  −0.01, *p* = 0.84) and competence (*β* =  0.02, *p* = 0.80) were not related to palliative knowledge. “Negative perception”, however, was negatively associated with palliative knowledge (*β* =  −0.20, *p* < 0.01).

**Conclusions:**

This study suggests continuing education to decrease nurses’ “negative perception” attitude regarding the provision of information to patients and families to provide better palliative and EOL care.

**Implications for Practice:**

Nurses’ attitudes and competences with respect to palliative care and EOL care are critical. Areas for further research and advanced palliative and EOL care-related education and training are suggested and may be applied in future clinical interventions.

## Introduction

Palliative care practices have developed rapidly in health care facilities in the past decade. The definition of palliative care has been extended; the concept of end-of life (EOL) care differs throughout the world, and nurses’ knowledge, attitudes, and contributions to EOL care vary (Badir et al., 2016; Langley et al., 2014; Wilson, Avalos & Dowling, 2016). In Taiwan, the related issue of not-for-resuscitation (NFR) orders has been debated for at least two decades, especially for patients with life-threatening illnesses. Under these circumstances, nurses usually have more interaction with palliative care patients than other health care workers do. Nurses are encouraged to provide the most appropriate information to help patients and families during critical situations; therefore, they need competence in caring for patients with life-threatening illnesses (Church, 2016; Fuoto & Turner, 2019) and in helping make the most appropriate decisions for patients. When health care professionals do not provide clear and adequate explanations of EOL care to patients and their families, the lack of information may impact their decisions (Cable-Williams et al., 2014; Johnstone et al., 2016; Sritharan et al., 2017).

Previous research has focused on nurses’ care delivery in intensive care units (Kao et al., 2013; Su et al., 2008; Wilson, Avalos & Dowling, 2016) and on caregiver stress regarding decision-making for the further treatment of patients (Kao et al., 2016). Limited Taiwanese research has mentioned nurses’ competence and knowledge regarding palliative and EOL care or discussed the issue of palliative and EOL education needs for nurses. Nurses usually focus on providing care to enhance patient comfort and reduce physical pain. However, they should also be knowledgeable about how to help patients and their families consider whether palliative and EOL care are appropriate. Effective palliative and EOL care training for nurses could positively impact their ability to provide high-quality care with respect, acceptance, and sincerity. Furthermore, little Taiwanese research has mentioned nurses’ attitudes toward providing palliative and EOL care and the relationship between these attitudes and nurses’ competence in providing care to EOL patients. For the current study, we used our previously-developed competence and attitude scale (Lin & Huang, 2014) to examine clinical nurses.

### Conceptual framework

According to a previous study (Ferrell, Malloy & Virani, 2015), a modified systematic review (Bassah, Seymour & Cox, 2014), and Bandura’s social cognitive theory (Bandura, 1986), performance and competence are correlated. Competence comprises the union of knowledge, skills, and attitudes in the performance of various activities. Especially for individuals in palliative care nursing, competence encompasses the comprehensive knowledge needed to care for both patients and their decision makers. We developed our competence measure according to Bandura’s learning theory (Bandura, 1986). It measures the respondent’s understanding of the professional capabilities needed to manage the patient’s and his or her family’s emotional reactions, deal with pain and symptom management, and manage stress (Coffey et al., 2016; Ersek, Kraybill & Hansberry, 1999).

Previous research has noted that inadequate confidence in nurses may influence the quality of palliative care they provide (Lippe et al., 2017). In particular, Pesut et al. (2014) have revealed positive effects of palliative care education interventions on nurses’ knowledge, abilities, and attitudes regarding care for dying people in several settings. However, the majority of studies included in the Pesut et al. (2014) meta-analysis were from the United Kingdom and America. Limited Taiwanese research has examined competence or attitudes in palliative and EOL care studies; the current research addresses this gap by examining Taiwanese nurses’ knowledge, competence and attitudes. In this study, nurses’ competence is defined as their confidence in responding to patients’ physical symptoms, emotional stress, grief and loss, collaboration with families, and legislative concerns. Nurses’ attitudes toward EOL are defined as their perceptions of providing care to people at the end of life in clinical practice.

The purposes of the present study were to (1) assess the palliative care knowledge, competence and attitudes of nurses in a general hospital and (2) examine the paths connecting nurses’ previous EOL experience, competence, attitude perception, and knowledge.

## Methods

### Study design

A cross-sectional study, purposive sampling, and a structured questionnaire survey were adopted to collect data at a general hospital in southern Taiwan. All the paper surveys were provided to eligible participants and put in an envelope. A structural equational modeling (SEM) approach was employed to analyze the path of EOL experience, competence, attitude perception, and knowledge.

### Sample

Nurses aged 20 years or older who had at least three months of professional experience in clinical nursing practice were eligible for the study. We recruited 682 nurses. The root mean square error of approximation (RMSEA) is commonly used as an index to assess the entire model data-model fit; the null hypothesis (H0), referred to as the model, fits well (i.e., close to 0), and the alternative hypothesis (H1), referred to as the model, fits inadequately (i.e., >.05). Given a model with degrees of freedom (*df*) =50, a desired power = 80%, and reasonable H1, the sample size is estimated to be 351 (Hancock & French, 2013).

### Ethical considerations

Ethical approval was provided by the Institutional Review Board of the E-Da hospital (EMRP-09106N). The researcher contacted eligible participants about the aims of the study, and each participant submitted a written consent form. All the participants filled out the instruments (paper-based) and put them in a provided envelope. The investigator collected all the data from different working units in the general hospital. Data were collected from August to December 2017.

### Instrumentation

Data for the survey study were gathered using instruments developed by Lin & Huang (2014). Several scales were used in this study to assess demographics; job characteristics; and knowledge, attitude, and competence, as follows:

### Demographic characteristics

The nurse demographic questionnaire included items related to age, gender, marital status, education, rank, work unit, and professional experience.

### Palliative care knowledge

The palliative care knowledge questionnaire was developed by Lin & Huang (2014) and is based on the definition of palliative and EOL care according to the medical condition, legal context in Taiwan, purpose, appropriate duration of care, etc. Four hospice experts (one psychiatric physician, one social worker, one advanced nursing specialist, and one measuring specialist) were invited to review the palliative care knowledge questionnaire to improve its content validity; then, the questionnaire was modified based on the reviewers’ opinions for construct validity. The final questionnaire includes 17 questions with three answer options, only one of which is correct. We totaled the scores for each participant and found that the higher the total score was, the greater the participant’s knowledge of palliative and EOL care was. The Cronbach’s *α* was .84 in this study.

### Nurses’ Palliative Attitude Scale

We adopted the Nurses’ Palliative Attitude Scale developed by Lin & Huang (2014) to identify how nurses perceive and interact with terminally ill patients regarding palliative care. Four hospice experts were invited to review the Nurses’ Palliative Attitude Scale to improve its content validity; then, the scale was modified based on the reviewers’ opinions for construct validity. The revised scale comprises 14 questions answered on a five-point Likert scale ranging from “1 = strongly disagree” to “5 = strongly agree”. The total score ranges from 14 to 70 points, with higher total scores indicating better attitudes toward palliative and EOL care. A Cronbach’s *α* of 0.77 was found for this study.

### Nurses’ Competence Scale

We used a scale that we developed ourselves (Lin & Huang, 2014) to evaluate nurses’ competence in palliative and EOL care. Four hospice experts were invited to review the Nurses’ Competence Scale to improve its content validity; then, the scale was modified based on the reviewers’ opinions for construct validity. The scale comprises 15 items answered on a five-point Likert scale in which “strongly disagree, disagree, neutral, agree, strongly agree” receive scores of 1–5. Total scores range from 15–75 points. The higher the total scores are, the greater the respondent’s competency is. The Cronbach’s α for the Chinese version was 0.93 in this study.

### Statistical analysis

The data were analyzed using SPSS (Statistical Package for the Social Sciences) version 18.0 and IBM SPSS Amos for Windows version 22.0. The data were screened for missing data, skewness and kurtosis for normality. Less than 1% of the data entries were missing, and they were corrected with the original paper-based questionnaires. For the 14-item attitude scale, two items (items 7 & 8) showed skewness values -.61 and -.74, two items (items 8 & 9) showed kurtosis values .94 and -.63; the remaining 12 items showed skewness and kurtosis values less than ±.5. For the 15-item competence scale, their skewness and kurtosis values were all less than ±.5. The results indicate symmetrical distribution. Furthermore, both the attitude and competence scales were five-category measurements, and maximum likelihood estimation was employed to assess the relationship among attitude, competence, and knowledge using SEM (Rhemtulla, Brosseau-Liard & Savalei, 2012). Descriptive statistics, i.e., frequencies, percentages, and averages, were utilized to report on the analyzed variables.

For validity testing of the measurement model, the total sample (*N* = 682) was randomly divided into two groups, Group 1 (N1 = 343) and Group 2 (N2 = 339). Group 1 was used to conduct exploratory factor analysis (EFA) for the two scales, the Nurses’ Palliative Attitude Scale and the Nurses’ Competence Scale. Based on the EFA results, confirmatory factor analysis (CFA) was applied to assess the psychometric components of the two scales in Group 2 (N2 = 339) and to examine the paths among the exogenous variables (i.e., previous palliative experiences, coursework), the mediators (i.e., nurses’ palliative care attitudes and competence), and the endogenous variable (i.e., palliative care knowledge). Statistical significance was set at *p* < 0.05. Several model fitness indices were employed to assess the fitness of the measurement model, including the normed chi-square (NC, *χ*^2^/df < 3.0), RMSEA 0.08−0.05, and comparative fit index (CFI) >0.95 (Kline, 2005).

## Results

### Demographic characteristics of the sample

All the participants (*n* = 682) were registered nurses, and the response rate was 97.8%. The studied characteristics of the participants included age, marital status, education, rank, work unit, and professional experience (Table 1). Most of the participants were female (*n* = 649, 95.2%); only 33 males participated (4.8%). The average age of the participants was 29.2 years, more than half were single, and 82.6% had a college degree. Regarding their rank at the hospital, the lowest level, N, accounted for the majority of all ranks (236, 34.6%). The nurses’ work units included the following: the general ward (*n* = 334, 49%) was the most common unit, followed by the intensive care unit (ICU; *n* = 162, 23.8%) and emergency room (ER; *n* = 59, 8.7%). The majority of the nurses had 3-5 years of professional experience (*n* = 176, 25.8%). Regarding coursework or experience related to palliative care (Table 1), most of the nurses had cared for patients or families dealing with NFR (*n* = 627, 91.9%), and the majority of the nurses had cared for palliative patients (*n* = 615, 90.2%) and had taken palliative care coursework (*n* = 667, 97.8%).

**Table 1:** Participants’ demographics , EOL care experiences, knowledge, attitudes, and competence (*N* = 682).

**Variables**	N	%	Mean	SD	Range
**Age**			29.2	6.1	20-48
**Gender**	649	95.2			
Female	33	4.8			
Male					
**Marital status**	527	77.3			
Single	155	22.7			
Married					
**Education**	119	17.4			
Associate’s degree	563	82.6			
≥BSN					
**Position**	658	96.5			
Registered Nurse	24	3.5			
Head Nurse					
**Rank**	236	34.6			
N	174	25.6			
N1	224	32.8			
N2	48	7.0			
N3					
**Prof. Exp.**	92	13.5			
<1 yr.	154	22.6			
1–2 yrs.	176	25.8			
3–5 yrs.	122	17.9			
7–10 yrs.	138	20.2			
>11 yrs.					
**Work unit**	59	8.7			
① ER	334	49.0			
② General Ward	20	2.9			
③ Psychiatric Ward	27	4.0			
④ Gyn/Pediatric Ward	162	23.8			
⑤ICU	36	5.3			
⑥ HR	7	1.0			
⑦ Oncology Ward	37	5.4			
⑧ Other					
**EOL experience**	615	90.2			
Yes					
**EOL care coursework**	667	97.8			
Yes					
**Time since EOL care coursework**	64	9.4			
①<1 month	108	15.8			
>1 month	204	29.9			
>3 months	221	32.4			
>6 months	85	12.5			
>12 months					
**EOL care knowledge**			76.10	9.91	31.25–100
**Palliative attitudes**			48.93	6.08	28–75
**Nurses’ self-competence in EOL care**			46.89	8.59	18–90

**Notes.**

BSN Bachelor of Science in Nursing. Prof. Exp. Professional Experience ER Emergency Room Gyn Gynecology ICU Intensive Care Unit HR Hemodialysis Room CEOL Caring for end-of-life patients.

### EOL knowledge, nurses’ palliative attitudes, and competence

Overall, the nurses achieved a correct answer rate of 76% on the *EOL* knowledge test (Table 2). Three items were answered correctly by less than 50% of the nurses: “Q1. Define palliative care (15%)”; “Q12. What treatments should physicians provide to terminally ill or dying patients or patients without vital signs?” (21%); and “Q4. Who cannot be a witness when signing the document of intent for palliative and end-of-life (EOL) care?” (46%). For the other questions, the rates of correct answers ranged from 70%∼100%. We then transformed the palliative knowledge scores to reflect a scale from 0 to 100, and the mean score was 76.1 (± 9.9); the range was 31.3 to 100 (Table 1). The EOL knowledge score significantly differed among work units (Table 3). As shown in Table 3, participants from the oncology ward reported the highest EOL knowledge score of 78.71 (±11.08), followed by participants from the emergency room (ER) with 78.71 (±8.22) and the general ward with 77.00 (±9.01). The lowest EOL knowledge score was from the hemodialysis room (HR) 73.78 (±9.78), followed by other wards 73.82 (±13.24) and gynecologic/pediatric wards 74.07 (±7.29).

**Table 2:** EOL knowledge scores of the participating nurses (*N* = 682).

***Question***		***Correct responses*%**
Q1	Define terminally ill patients.	15%
Q12	What treatments should physicians provide for terminally ill or dying patients or patients without vital signs?	21%
Q4	Who CANNOT be a witness when signing the letter of intent for hospice palliative care?	46%
Q7	What qualifications must a physician have to make a diagnosis of a terminally ill patient without cardiopulmonary resuscitation?	70%
Q9	Who should make a declaration to countermand the intent for hospice palliative care?	74%
Q16	Can a minor child of a terminally ill patient sign the letter of intent for hospice palliative care?	77%
Q11	Which item is included in the Hospice Palliative Care Act?	79%
Q5	How many witnesses are required for signing the letter of intent for hospice palliative care?	85%
Q15	If a terminally ill patient has become unconscious or failed to clearly express his/her will, his/her close relative may sign consent in his/her place. The consent should express the patient’s will before becoming unconscious.	85%
Q6	How many doctors are needed to approve the terminally ill patient to allow cardiopulmonary resuscitation to be refused?	86%
Q17	Is hospice palliative care equal to euthanasia?	87%
Q14	For a terminally ill patient to write a letter of intent for hospice palliative care, what entitles him/her to the legal capacity to make this declaration, and what should his/her minimum age be?	88%
Q10	In addition to the original undersigned individual, which hospital committee should approve the termination or withdrawal of CPR?	91%
Q3	The Hospice Palliative Care Act does not apply to…	93%
Q13	What is cardiopulmonary resuscitation?	93%
Q2	Who must sign the letter of intent for “Not for Resuscitation” (NFR) status when a terminally ill patient is still conscious?	96%
Q8	When a terminally ill patient become unconscious or has failed to express his/her wishes, who should submit the NFR agreement?	100%

**Table 3:** Nurses’ EOL knowledge, attitude, and competence by work unit.

Work unit	EOL care knowledge mean (SD)	Palliative attitude mean (SD)	Nurses’ competence in EOL care mean (SD)
① ER (*n* = 59)	78.71 (8.22)	48.61 (6.40)	48.24 (7.37)
② General Ward (*n* = 334)	77.00 (9.61)	49.20 (6.19)	47.17 (8.63)
③ Psychiatric Ward (*n* = 20)	75.00 (11.11)	48.75 (6.09)	42.90 (10.64)
④ Gyn/Pediatric Ward (*n* = 27)	74.07 (7.29)	46.11 (1.26)	43.07 (7.91)
⑤ICU (*n* = 162)	74.61 (10.09)	48.77 (6.04)	47.90 (8.30)
⑥ HR (*n* = 36)	73.78 (9.78)	48.89 (4.24)	43.28 (9.16)
⑦ Oncology Ward (*n* = 7)	80.36 (11.08)	52.29 (7.63)	53.86 (6.87)
⑧ Other (*n* = 37)	73.82 (13.24)	49.32 (5.48)	46.89 (8.59)
F value	2.48	1.29	3.94
*p* value	.02	.25	<.001

**Notes.**

ER Emergency Room Gyn Gynecology ICU Intensive Care Unit HR Hemodialysis Room CEOL Caring for end-of-life patients

The average score on the Nurses’ Palliative Attitude Scale was 48.93 (SD 6.08), with a range from 28 to 75. Scores on the Nurses’ Palliative Attitude Scale did not significantly differ among work units (Table 3). As shown in Table 3, participants from the oncology ward reported the highest attitude score of 52.29 (±7.63), followed by other wards 49.32 (±5.48) and the general ward 49.20 (±6.19). The lowest attitude scores were from the gynecologic/pediatric ward 46.11 (±1.26), ER 48.61 (±6.40) and psychiatric ward 48.75 (±6.09).

The average score on the Nurses’ Competence Scale was 46.89 (SD 8.59), with a range from 18 to 90. Scores on the Nurses’ Competence Scale significantly differed among work units (Table 3). As shown in Table 3, participants from the oncology ward reported the highest attitude score of 53.86 (±6.87), followed by the ER 48.24 (±7.37) and the general ward 47.17 (±8.63). The lowest attitude scores were from the psychiatric ward 42.90 (±10.64), gynecologic/pediatric ward 43.07 (±7.91) and HR 43.28 (±9.19).

### Relationships among knowledge, attitudes and demographic characteristics

In Table 4, nurses’ professional experience is positively correlated with their position, professional level (rank), EOL care experience, competence in EOL, and other knowledge. Nurses who have a positive attitude seem more likely to have more competence in dealing with patients’ symptoms at EOL and better knowledge of EOL care (*r* = 0.49, *P* < 00.001). The more palliative knowledge nurses had, the more competence (*r* = 0.24, *P* < 00.001) they felt. Moreover, competence dealing with patients’ symptoms in EOL care was correlated with older nurses (*r* = 0.19, *P* < 0.001).

**Table 4:** Correlation of variables (*N* = 682).

Variables	1	2	3	4	5	6	7
1. Age	1.00						
2. Professional Exp.	0.85^***^	1.00					
3. Rank	0.57^***^	0.66^***^	1.00				
4. EOL Exp.	0.17^***^	0.19^***^	0.18^***^	1.00			
5. Attitude	0.05	0.06	0.02	0.10^*^	1.00		
6. Competence	0.19^***^	0.12^**^	0.04	0.07	0.49^***^	1.00	
7. Knowledge Test	0.05	0.12^**^	0.12^**^	0.12^**^	0.10^**^	0.24^***^	1.00

**Notes.**

* *P* < .05

** *P* < .01

*** *P* < .001

Professional Exp. Professional experience EOL Exp. End-of-life experiences

Factor analysis of this attitude scale yielded a Kaiser-Meyer-Olkin (KMO) value of 0.76, and Bartlett’s test yielded a value of 2742.53, *df* = 91, *p* <  0.001, supporting the appropriateness of EFA. According to the scale content, two factors, i.e., “positive perception” and “negative perception” toward palliative care, were proposed. Ten items were related to “positive perception”, and four items were related to “negative perception”. For the competence scale, the KMO was 0.76, and Bartlett’s test yielded a value of 5337.07, *df* = 105 (*P* < .001), sufficient for conducting EFA. According to the scale content, a two-factor measurement model with 15 items was proposed and tested using SEM. Nine items (1–9) were related to the “legislative competence” factor, and six items (10–15) were related to the “professional care competence” factor.

The Group 1 sample was randomly selected to explore the hypothesized measurement model. The results for the original measurement model revealed inadequate model fit. Six items from the positive perception factor and one item from the negative perception factor showed factor loadings <.6, and these items were removed; they were: “1) I would not feel uncomfortable discussing the condition of a terminally ill patient with other healthcare professionals”; “7) I would be willing to sign an NFR order for my family, if necessary”; “8) I would be willing to sign an advance directive for palliative and hospice care”; “12) I need to acquire more knowledge of palliative and hospice care”; “13) I feel comfortable sharing my experience in palliative and hospice care with other healthcare professionals”; and “14) I feel comfortable sharing my experience in palliative and hospice care with relatives and friends.” Three items showed adequate factor loadings (.74–.87) with the positive perception factor: “2) I would not feel uncomfortable discussing death and dying with families of terminally ill patients”; “3) I would not feel uncomfortable discussing death and dying with terminally ill patients”; “4) I would not feel uncomfortable assisting families of terminally ill patients in submitting an NFR order”; and “5) I would not feel uncomfortable assisting terminally ill patients in submitting an NFR order.”

One item with a factor loading <.60 was removed from the negative perception factor; the item was “6) I would be afraid to tell my family member that he/she were dying.” The remaining three items of the negative perception factor showed factor loadings of .64 - .93, and they were: “9) I would feel upset all day long if I provided care to terminally ill patients”; “10) I feel suffocated all day long if I provide care to terminally ill patients”; and “11) I feel deeply grieved if a terminally ill patient whom I care for is approaching death.”

**Figure 1:**
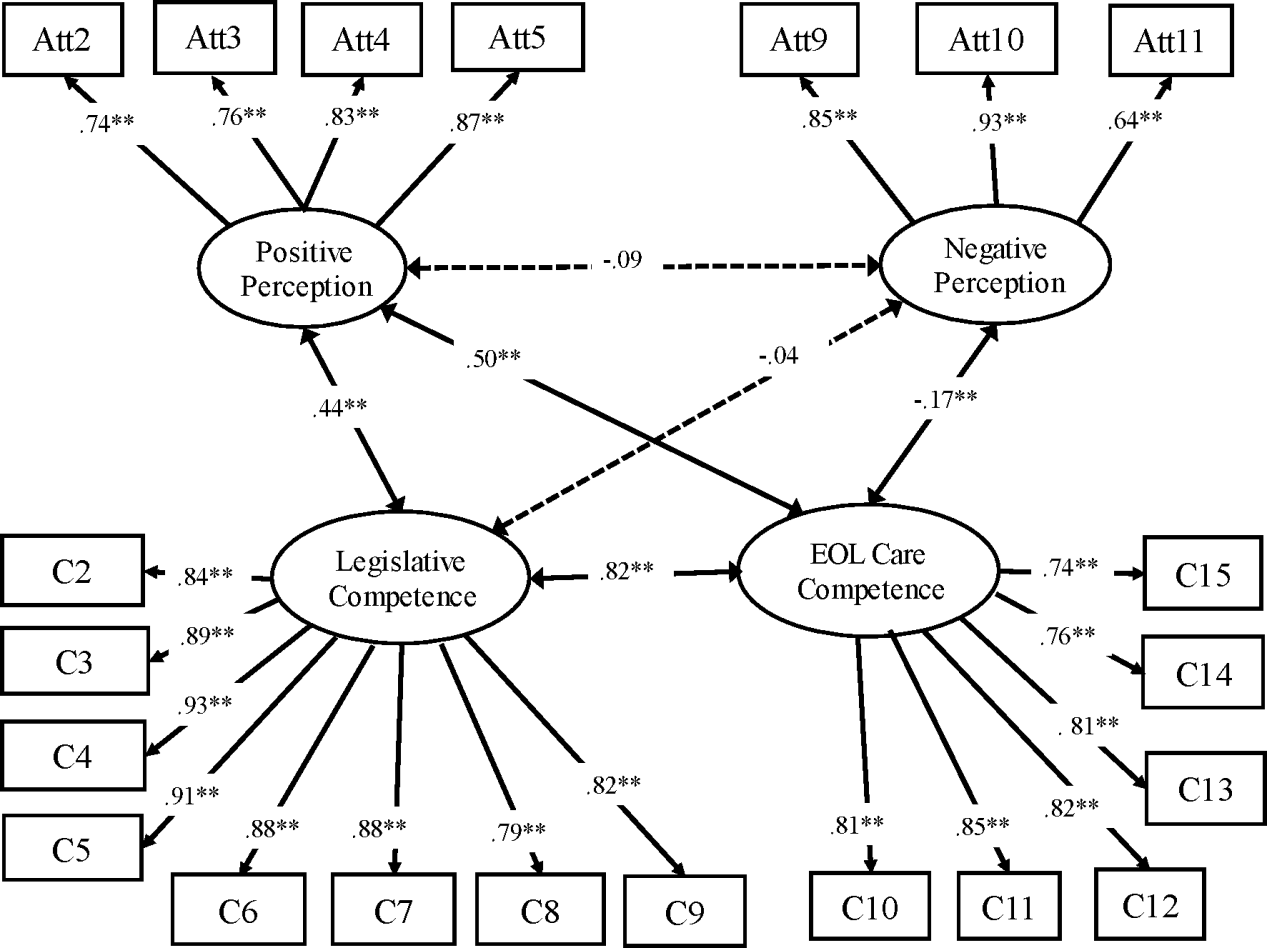
The measurement model of the Nurses’ Palliative Attitude Scale and the Nurses’ Self-competence Scale (Group 1, *N* = 343). Model fitness summaries: *χ*^2^ = 409.60, *df* = 167; CFI = 0.96; RMSEA = 0.07. ^∗^*p* < 0.05, ^∗∗^*p* < 0.01. Double-arrowh.

One item from the “legislative competence” factor that showed a factor loading <.6 was removed; it was “I can explain the fundamental concept of hospice and palliative legislation to others.” The remaining eight items of the legislative competence factor assessed nurses’ perceived self-competence in legislative aspects of hospice and palliative care. For example, item 2 was, “I would be able to respond to concern about advance directives for people and their families.” The other factor, “EOL care competence”, contained six items to assess nurses’ perceived competence in EOL care, such as physical problems, family conflicts, and grieving. The final EFA model consisted of a two-factor attitude scale with 7 items and a two-factor competence scale with 14 items (Fig. 1). The final measurement model had acceptable model fit indices (*χ*^2^ = 458.67, *df* = 201, *p*<0.001, NC = 458.67/201 = 2.28, RMSEA = 0.06, and CFI = 0.96).

### Path analysis: attitudes, competence, and knowledge

**Figure 2:**
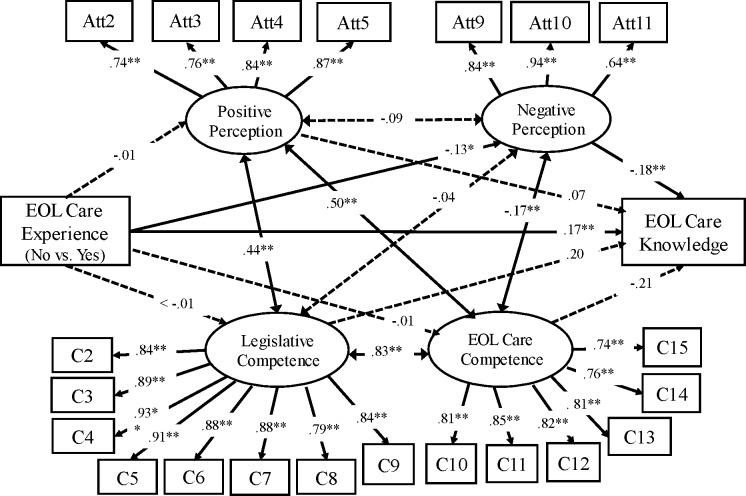
Path model: palliative attitudes, competence, and knowledge (Group 1, *N* = 343). Model fitness summaries: *χ*^2^ = 458.67, *df* = 201; CFI = 0.96; RMSEA = 0.06. ∗*p* < 0.05, ^∗∗^*p* < 0.01. Double-arrowhead line: correlation coefficient. One-ar.

Path analysis was employed to examine the paths among nurses’ attitudes, competences, and knowledge regarding palliative care. EOL care experience was a significant variable associated with nursing knowledge of palliative care. Nurses who reported experience caring for palliative patients obtained higher EOL care knowledge scores than those without such experience (76.60 vs. 71.55, *p* < .01). As seen in Fig. 2, “negative perception” attitude was negatively associated with EOL care knowledge (*β* =  − .18, *p* = .02) in Group 1. However, “positive perception” attitude, “legislative competence”, and “EOL care competence” were not significantly associated with EOL care knowledge. EOL care experience was negatively associated with “negative perception” attitude (*β* =  − .12, *p* < .01) and positively associated with EOL care knowledge (*β* = .17, *p* < .01).

**Figure 3:**
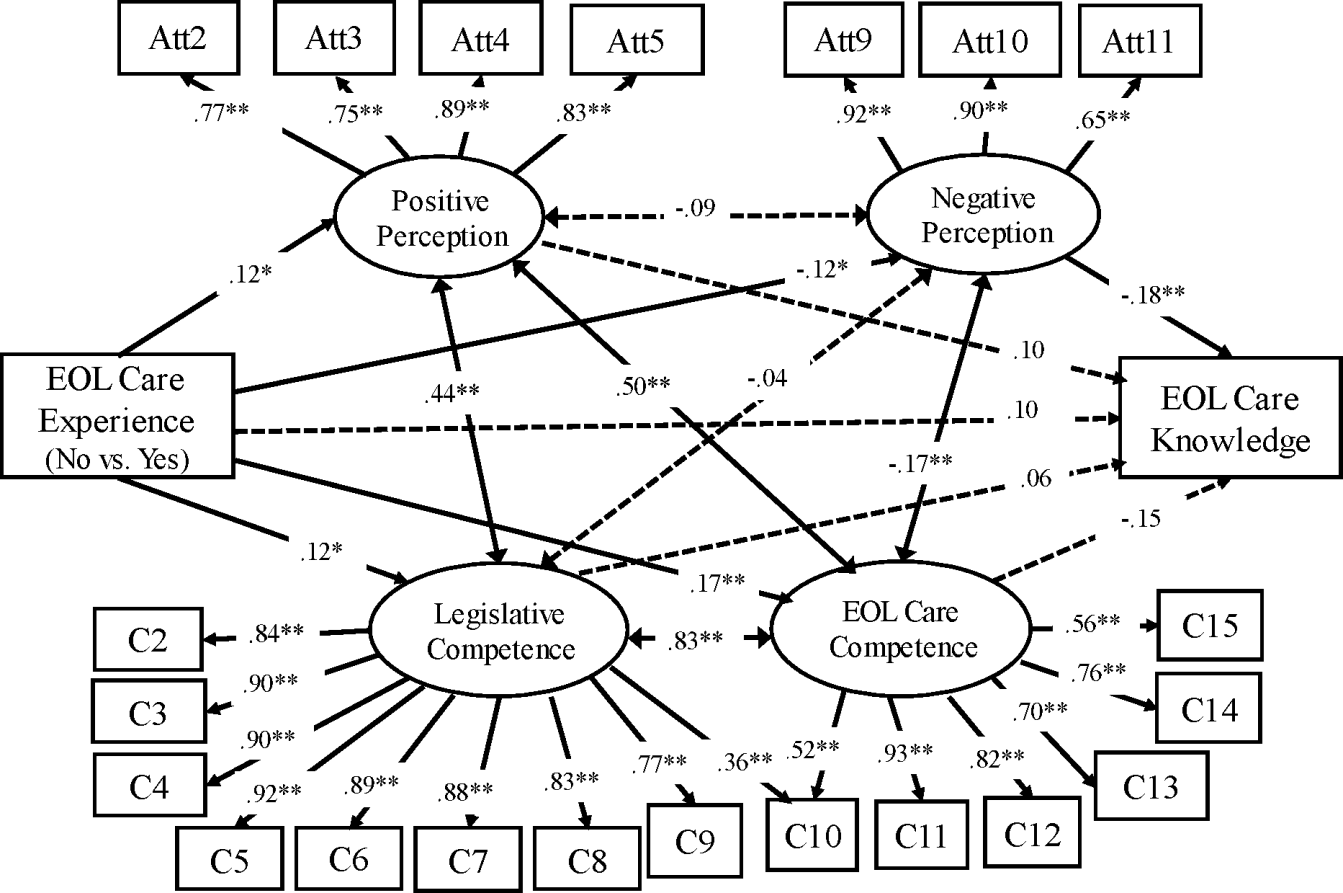
Path model: palliative attitudes, self-competence, and knowledge (Group 2, *N* = 339). Model fitness summaries: *χ*^2^ = 489.28, *df* = 202; CFI = 0.96; RMSEA = 0.07. ^∗^*p* < 0.05, ∗∗*p* < 0.01. Double-arrowhead line: correlation coefficient. O.

As shown in Fig. 3, EOL care experience was positively associated with “positive perception,” “legislative competence,” and “EOL care competence” (*β* = .12, *p* = .03; *β* = .12, *p* = .04; *β* = .17, *p* = <.01). On the other hand, EOL care experience was negatively associated with “negative perception” attitude (*β* =  − .12, *p* = .03) in Group 2. In contrast to the path result in Group 1, one item on the competence scale (Question 10) was related to both the “legislative competence” and “EOL care competence” factors; the factor loadings were .36 and .52, respectively. Question 10 was “I would be able to resolve conflicts in EOL care preferences within the family for a terminally ill patient.” Finally, the structure of the measurement model was similar in both Groups 1 and 2, including with respect to the factor loadings and model fit indices.

## Discussion

This study examined the knowledge, attitudes, and competence of nurses in providing palliative and EOL care. Several important results of this study are as follows: (1) a description of the palliative care knowledge, competence and attitudes of nurses at a general hospital; (2) the psychometric components, i.e., positive and negative perceptions of nurses’ palliative attitudes, are constructed in the EFA measurement model; (3). relationships are identified among previous experience, attitude perceptions, competence, and knowledge in the CFA path models.

First, regarding the palliative care knowledge test, only 76% of the questions were answered correctly, which supports the need for continuing education that furnishes the knowledge nurses need to provide qualified EOL care. In Taiwan, although families need support and treatment information to make appropriate decisions for their ill loved ones, the determination that a patient is at the EOL stage is usually made by physicians. Although an NFR may be considered, it is a sensitive issue in Chinese culture; therefore, physicians may not always suggest it as an option. Under these circumstances, who is obligated to provide families or patients with further information to help them decide between treatment and palliative care? Nurses’ competence and attitudes should be improved to enhance the quality of palliative and EOL care.

Second, our findings suggest that nurses’ attitudes could be categorized as positive and negative perceptions toward palliative attitudes. Positive perception is weakly negatively correlated with negative perception and moderately positively correlated with nurses’ competence. Negative perception attitude is not significantly correlated with nurses’ competence. Positive perception attitudes are more important than negative perception attitudes in contributing to nurses’ competence.

Third, our results suggest that nurses’ competence is not significantly related to palliative knowledge, which is in contrast to our hypotheses. Of several predictors, nurses’ negative perceptions and previous experience caring for EOL patients were significantly related to their knowledge. Our study showed that nurses who had palliative and EOL care experience had better attitudes toward EOL care than those without experience, which was consistent with a previous study (Wilson, Avalos & Dowling, 2016). However, in the path model, the previous experience of EOL patients is negatively related to negative perception, neither positive perception nor competence. The literature reveals that professional experience is associated with competence and knowledge. For example, a previous study noted that the age and professional experience of health care providers may affect their attitudes toward NFRs (Su et al., 2008). Similarly, Coffey et al. (2016) suggest that older nurses with longer durations of professional experience feel more confident about managing patients’ symptoms and providing EOL care. Knowledgeable, highly self-competent, experienced nurse professionals can provide appropriate information to help terminally ill patients and their families make appropriate decisions.

Additionally, nurses with attitudes characterized as “negative perception” were more likely to lack palliative care knowledge and may need more continuing education to manage the challenges of caring for EOL patients. This explains why a “negative perception” attitude in nurses significantly impacted nurses’ knowledge. Since the measurement of attitude was developed from Lin & Huang (2014) and has not been used in related research, there are no results available for comparison.

### Limitations

This study was conducted at a local general hospital, and the findings cannot be generalized to other hospitals. All the questionnaires were developed by Lin & Huang (2014). The questionnaires considered knowledge and competence regarding palliative and EOL care, and “advance directives” or “advance care planning” may be added to the content to provide more comprehensive information for terminally ill patients or their relatives. Other variables may need to be added to evaluate interest in increasing knowledge of and needs related to palliative care among patients and nurses at a general hospital. Additionally, a lack of standardized guidelines for hospitals suggests a need to develop specific, standardized guidelines for palliative and EOL care and to evaluate the quality of care for EOL patients. Moreover, definitions of advance directives and NFRs are lacking in continuing education, which has led to some vague perceptions and unclear impacts on clinical practice. Moreover, the focus of the present study could have been expanded to include physicians, other health care professionals, or informal caregivers.

## Conclusion

Nurses who have received continuing education related to palliative care had higher scores on the knowledge test, reflecting the effectiveness of continuing education. Future palliative care education programs for health care providers should include sharing palliative care experiences, discussing principles and legislation, determining appropriate referrals, and promoting nurses’ competence in caring for palliative and EOL patients. Furthermore, the development of standardized guidelines for EOL care in all hospital settings should be considered.

Administrators need to promote knowledge of palliative and EOL care among nurses to improve nurses’ competence in providing qualified palliative care. Recently, efforts to improve palliative care in hospitals and health care facilities have increased. However, while expertise in palliative care is increasing, unmet needs remain.

### Implications for nursing

The attitude and competence scales used in this study could be used in different health care settings to determine the educational needs of nurses. These brief scales can help identify nurses’ educational needs in various health centers. This study supports several preliminary examinations of the differences in palliative care competence at the participating local health center. Areas for further research, advanced palliative care-related education and training are suggested and may be applied to future clinical interventions.

##  Supplemental Information

10.7717/peerj.11864/supp-1 Supplemental Information 1 Raw data

10.7717/peerj.11864/supp-2 Supplemental Information 2 Data Code Book

10.7717/peerj.11864/supp-3 Supplemental Information 3 Questionnaire in Chinese

10.7717/peerj.11864/supp-4 Supplemental Information 4 Questionnaire in English

## Additional Information and Declarations

### Data Availability

The following information was supplied regarding data availability: The data is available in the Supplemental Files.
