# Supplementary material for: Nurses’ knowledge, attitude, and competence regarding palliative and end-of-life care: a path analysis
Source: PeerJ. 2021 Jul 26;9:e11864. doi: 10.7717/peerj.11864 (PMC8320516; doi:10.7717/peerj.11864)
Supplement: Supplemental Information 2 [file peerj-09-11864-s002.docx]

Title: Nurses’ knowledge, attitude, and self-competence regarding palliative end-of-life care: A path analysis

**Data code book**

| Variable name | Label | Value |
| --- | --- | --- |
| Gender | Sex | 1 Male  2 Female |
| Marital | Marital status | 1 Single  2 Married |
| Education | The highest degree in nursing | 1 Associate’s degree  2 BSN (bachelor of science in nursing) or higher |
| Position | Nursing job title | 1 Registered nurse  2 Head nurse |
| Rank | The level in nursing practice | 1 N, novice or entry level in nursing practice  2 N1, N1 level in nursing practice  3 N2, N2 level in nursing practice  4 N3, N3 level in nursing practice  5 N4, N4 level in nursing practice |
| Prof. Exp. | Years of professional experience in nursing | 1 < 1 year  2 1–2 year  3 3–5 year  4 7–10 year  5 >11 year |
| Work unit | Working unit | 1 Emergency room  2 General ward  3 Psychiatric ward  4 Gyn and Pediatric ward  5 Intensive care unit  6 Hemodialysis room  7 Oncology ward  8 Others |
| EOL experience | Previous experiences in providing care to patients in the end of their lives | 0 No  1 Yes |
| EOL care coursework | Previous coursework regarding end-of-life care | 0 No  1 Yes |
| Time since EOL care coursework | Time since the last coursework about end-of-life care | 1 <1 Month  2 >1 Month  3 >3 Months  4 >6 Months  5 >12 Months |
