# Supplementary material for: Nurses’ knowledge, attitude, and competence regarding palliative and end-of-life care: a path analysis
Source: PeerJ. 2021 Jul 26;9:e11864. doi: 10.7717/peerj.11864 (PMC8320516; doi:10.7717/peerj.11864)
Supplement: Supplemental Information 3 [file peerj-09-11864-s003.doc]

**基本資料**

1. 性別：*^1^*⭘男 *^2^*⭘女
2. 年齡： 歲
3. 婚姻狀況：*^1^*⭘未婚；*^2^*⭘已婚；*^3^*⭘離婚；*^4^*⭘喪偶；*^5^*⭘分居
4. 宗教信仰：*^1^*⭘無；*^2^*⭘佛教；*^3^*⭘天主教；*^4^*⭘基督教；*^5^*⭘道教；*^6^*⭘其他
5. 教育程度：*^1^*⭘專科；*^2^*⭘大學(含二技、四技)；*^3^*⭘碩士或以上
6. 目前工作單位：*^1^*⭘急診；*^2^*⭘內科病房 科；*^3^* ⭘外科病房 科；*^4^*⭘精神科病房；*^5^*⭘婦產科病房；*^6^*⭘小兒科病房；*^7^*⭘ NSICU；*^8^*⭘MICU；

*^9^*⭘RCC；*^10^*⭘ CCU；*^11^*⭘ 外科加護病房；*^12^*⭘ PICU；*^13^*⭘洗腎室；

*^14^*⭘腫瘤科病房; *^15^*⭘安寧病房; *^16^*⭘其他

1. 工作總年資：*^1^*⭘＜1年內；*^2^*⭘1-2；*^3^*⭘3-4；*^4^*⭘5-6；*^5^*⭘7-10；*^6^*⭘＞11年
2. 工作職稱：*^1^*⭘護士；*^2^*⭘護理師；*^3^*⭘主管；*^4^*⭘專科護理師
3. 職務等級：*^1^*⭘N；*^2^*⭘N1；*^3^*⭘N2；*^4^*⭘N3；*^5^*⭘N4
4. 是否有照顧已簽屬DNR之生命末期病人的經驗 *^1^*⭘無；*^2^*⭘有
5. 是否有照顧過臨終病人的經驗 *^1^*⭘無；*^2^*⭘有
6. 在校期間是否曾上過安寧緩和療護的課程 *^1^*⭘無；*^2^*⭘有
7. 工作期間是否曾上過安寧療護相關的在職教育課程 *^1^*⭘無；*^2^*⭘有
8. 最近一次上的安寧相關課程距離現在有多久？

*^1^*⭘1個月內；*^2^*⭘1個月以上；*^3^*⭘3個月以上；*^4^*⭘半年以上；*^5^*⭘1年以上

**1.**知識

| ( ) | 1.末期病人是指： (1)近期內即將死亡者；(2)不超過六個月壽命者；(3)  不超過ㄧ年壽命者 |
| --- | --- |
| ( ) | 2.末期病人意識清醒時由誰簽署「不施行心肺復甦術意願書」？  (1)病人本人；(2)病人家屬；(3)病人朋友。 |
| ( ) | 3.安緩條例不適用於：(1)癌症病人；(2)肺炎病人；(3)漸凍病人。 |
| ( ) | 4.安緩醫療意願書之簽署，何人不得為見證人？ (1)病人家屬； (2)實施安寧緩和醫療之醫療機構之所屬人員；(3)病人朋友。 |
| ( ) | 5.安緩醫療意願書之簽署，在場見證至少需要幾人？  (1)一人；(2)二人；(3)三人。 |
| ( ) | 6.診斷為不施行心肺復甦術之末期病人，醫師應有幾位？  (1)一人；(2)二人；(3)三人。 |
| ( ) | 7.前條所指診斷，醫師需要何種資格？  (1)一般科別；(2)腫瘤專科；(3)相關專科。 |
| ( ) | 8.末期病人意識昏迷或無法清楚表達意願時，由誰出具「不施行心肺復  甦術同意書」？(1)病人朋友；(2)最近親屬；(3)同居人。 |
| ( ) | 9.欲撤除安緩醫療意願，何人必須出面聲明？  (1)病人朋友；(2)最近親屬；(3) 本人。 |
| ( ) | 10.心肺復甦術之終止或撤除，除了原簽署人之外，尚須醫療機構的何種  委員會通過？(1)「人事評議」；(2)「醫學倫理」；(3)「醫療工作」。 |
| ( ) | 11.何項內容包含於安緩醫療條例之中？  (1)不施行心肺復甦術；(2)放棄積極性治療；(3)放棄所有治療。 |
| ( ) | 12.對簽署安緩醫療意願書之末期病人於臨終、瀕死、或無生命徵象醫師需給予何種治療？(1)緩和性、支持性治療；(2)急救藥物；(3)視意願書內容而定。 |
| ( ) | 13.心肺復甦術指的是(1)氣管內插管及急救藥物注射；(2)體外心臟按壓、心臟電擊、心臟人工調頻、人工呼吸或其他救治行為；(3)以上皆是。 |
| ( ) | 14.預立安緩醫療意願書末期病人必須是完全行為能力者而且年紀？(1)十八歲以上；(2)**二十**歲以上；(3)三十歲以上。 |
| ( ) | 15.末期病人意識昏迷時，最近親屬可代為出具安緩醫療意願書，它與昏迷前**明示**意思必須是(1)相同；(2)相反(3)無關。 |
| ( ) | 16.未成年末期病人是否可簽署安緩醫療意願書？  (1)並無任何限制；(2)只能法定代理人；(3)可以且經法定代理人同意。 |
| ( ) | 17.「安緩醫療」等於「安樂死」嗎？(1)完全正確；(2)部分正確；(3)完全錯誤。 |

**2.態度**

|  | 護理人員態度量表題目 | **☺☺** | **☺** | **😐** | **☹** | **☹☹** |
| --- | --- | --- | --- | --- | --- | --- |
| 1. | 與醫療團隊討論末期病人我不會為難。 | **○** | **○** | **○** | **○** | **○** |
| 2. | 與末期病人之家屬談論死亡議題我不會為難 | **○** | **○** | **○** | **○** | **○** |
| 3. | 與末期病人談論死亡議題我不會為難。 | **○** | **○** | **○** | **○** | **○** |
| 4. | 協助末期病人*親屬*提出DNR我不會為難。 | **○** | **○** | **○** | **○** | **○** |
| 5. | 要求末期病人*本人*提出DNR我不會為難。 | **○** | **○** | **○** | **○** | **○** |
| 6. | 我不敢告知末症親屬近將死亡。 | **○** | **○** | **○** | **○** | **○** |
| 7. | 必要時，我會願意簽署親人的DNR。 | **○** | **○** | **○** | **○** | **○** |
| 8. | 必要時，我會願意簽署安緩醫療意願書。 | **○** | **○** | **○** | **○** | **○** |
| 9. | 照顧末期病人讓我終日不安。 | **○** | **○** | **○** | **○** | **○** |
| 10. | 陪顧臨終病人我會整天感到窒悶。 | **○** | **○** | **○** | **○** | **○** |
| 11. | 照護之病人臨至亡故我會感到哀傷不已。 | **○** | **○** | **○** | **○** | **○** |
| 12. | 我亟待充實安緩療護的相關知識。 | **○** | **○** | **○** | **○** | **○** |
| 13. | 我不吝與*醫療團隊*分享安緩療護的經驗。 | **○** | **○** | **○** | **○** | **○** |
| 14. | 我不吝與*親朋好友*分享安緩療護的經驗。 | **○** | **○** | **○** | **○** | **○** |

**3.自評能力**

|  | 護理人員能力自評量表 | **☺☺** | **☺** | **😐** | **☹** | **☹☹** |
| --- | --- | --- | --- | --- | --- | --- |
| 1. | 我能夠詮釋安緩醫療的立法精神。 | **○** | **○** | **○** | **○** | **○** |
| 2. | 我能夠答覆安緩醫療意願的*預立*問題。 | **○** | **○** | **○** | **○** | **○** |
| 3. | 我能夠答覆安緩醫療意願的*見證*問題。 | **○** | **○** | **○** | **○** | **○** |
| 4. | 我能夠答覆安緩醫療醫師的*資格*問題。 | **○** | **○** | **○** | **○** | **○** |
| 5. | 我能夠答覆安緩醫療意願的*代立*問題。 | **○** | **○** | **○** | **○** | **○** |
| 6. | 我能夠答覆安緩醫療意願的*親等*問題。 | **○** | **○** | **○** | **○** | **○** |
| 7. | 我能夠答覆安緩醫療意願的*撤除*問題。 | **○** | **○** | **○** | **○** | **○** |
| 8. | 我能夠答覆違反安緩條例的*法律*問題。 | **○** | **○** | **○** | **○** | **○** |
| 9. | 我能夠答覆安緩醫療的文件保存問題。 | **○** | **○** | **○** | **○** | **○** |
| 10. | 我能夠疏解末期病人親屬的意願矛盾。 | **○** | **○** | **○** | **○** | **○** |
| 11. | 我能夠克服照護末期病人的情緒壓力。 | **○** | **○** | **○** | **○** | **○** |
| 12. | 我能夠克服面臨*病人*臨終的哀傷情緒。 | **○** | **○** | **○** | **○** | **○** |
| 13. | 我能夠克服面臨*親人*臨終的哀傷情緒。 | **○** | **○** | **○** | **○** | **○** |
| 14. | 我能夠冷靜面對末期病人的生理問題。 | **○** | **○** | **○** | **○** | **○** |
| 15. | 我能夠將所學的運用在家人身上。 | **○** | **○** | **○** | **○** | **○** |
