# Supplementary material for: Nurses’ knowledge, attitude, and competence regarding palliative and end-of-life care: a path analysis
Source: PeerJ. 2021 Jul 26;9:e11864. doi: 10.7717/peerj.11864 (PMC8320516; doi:10.7717/peerj.11864)
Supplement: Supplemental Information 4 [file peerj-09-11864-s004.docx]

**Information Sheet**

1. Gender: Male _____ Female ____

2. Age in Years: _____

3. Marital Status: (Choose one is more likely your situation)

(1). Single, not married _____ (2). Married/Cohabiting _____

(3). Divorced ______(4). Widowed ______(5)Separated______

4. Religion

(1) None_____(2).Buddhist _____ (3).Catholic ______(4).Christian ______

(5)Taoist _____ (6) Other ______

5. Education: (1)College/AD. Graduation ____ (2) College degree (4 years) ____

(3) Graduate School ______

6.Working unit：*^1^*ER；*^2^*Ward ；*^3^*Psych Ward；^4^GYN Ward；*^5^*⭘Pediatric Ward；*^6^* NSICU；*^7^*MICU；*^8^*RCC；*^9^* CCU；*^10^* SICU；*^11^*PICU；*^12^*HR；*^13^*Oncology ^14^Palliative Ward; *^15^* others

7.Professional experiences：

*^1^*⭘＜1year；*^2^*⭘1-2；*^3^*⭘3-4；*^4^*⭘5-6；*^5^*⭘7-10；*^6^*⭘＞11yesrs

8.position：*^1^*AD；*^2^*RN；*^3^*head nurse；*^4^*practitioner

9.Job Level：*^1^*⭘N；*^2^*⭘N1；*^3^*⭘N2；*^4^*⭘N3；*^5^*⭘N4

10. Have previous experience in taking care of undersigned DNR terminal patient? *^1^*⭘No；*^2^*⭘Yes

11. Have previous experience in taking care of terminal patient? *^1^*⭘No；*^2^*⭘Yes

12.Have taken Hospice Palliative Care course in School?

*^1^*⭘ No；*^2^*⭘ Yes

13. Have been received continuing education about Hospice Palliative Care course during work? *^1^*⭘ No；*^2^*⭘Yes

1. How long have it been since last tine taking Hospice Palliative Care course

*^1^*< 1 month；*^2^*1~3 months；*^3^* 3~6 months；*^4^* 6~12 months；*^5^* >12 months

1. **Knowledge**

***Question***

1. The definition of terminally ill patients.

2. Who must sign the letter of intent for “Not for Resuscitation” (NFR) status when a terminally ill patient is still conscious?

3. The Hospice Palliative Care Act does not apply to…

4. Who CANNOT be a witness when signing the letter of intent for hospice palliative care?

5. How many witnesses are required for signing the letter of intent for hospice palliative care?

6. How many doctors are needed to approve the terminally ill patient to allow cardiopulmonary resuscitation to be refused?

7. What qualifications must a physician have to make a diagnosis of a terminally ill patient without cardiopulmonary resuscitation?

8. When a terminally ill patient become unconscious or has failed to express his/her wishes, who should submit the NFR agreement?

9. Who should make a declaration to countermand the intent for hospice palliative care?

10. In addition to the original undersigned individual, which hospital committee should approve the termination or withdrawal of CPR?

11. Which item is included in the Hospice Palliative Care Act?

12. What treatments should physicians provide for terminally ill or dying patients or patients without vital signs?

13. What is cardiopulmonary resuscitation?

14. For a terminally ill patient to write a letter of intent for hospice palliative care, what entitles him/her to the legal capacity to make this declaration, and what should his/her minimum age be?

15. If a terminally ill patient has become unconscious or failed to clearly express his/her will, his/her close relative may sign consent in his/her place. The consent should express the patient’s will before becoming unconscious

16. Can a minor child of a terminally ill patient sign the letter of intent for hospice palliative care?

17. Is hospice palliative care equal to euthanasia?

| **2. Attitude** |
| --- |
| 1. I won't be embarrassed to discuss terminal patients with the medical team. |
| 2. I won't be embarrassed to talk to the families of the terminally ill about death |
| 3. I won't be embarrassed to talk to terminally ill patients about death. |
| 4. I won't be embarrassed to assist relatives of terminally ill patients with DNR. |
| 5. I won't be embarrassed to ask the terminal patient himself to ask for a DNR. |
| 6. I dare not tell my terminally ill relatives that he will die. |
| 7. If necessary, I will be willing to sign a loved one's DNR. |
| 8. If necessary, I will be willing to sign a medical will. |
| 9. Caring for the terminally ill makes me uneasy all day long. |
| 10. Accompanying the dying patients I will feel suffocated all day long. |
| 11. I will feel sad when the patient in care dies. |
| 12. I urgently need to enrich the knowledge of the treatment. |
| 13. I'm not shy about sharing my experience with the medical team. |
| 14. I don't hesitate to share the experience of treatment with friends and family. |

3. **Self- Competence**

| 1. I can explain the legislative spirit of health care. |
| --- |
| 2. I was able to answer the pre-set questions about the willingness to ease the medical  care. |
| 3. I was able to answer the testimony question of the willingness to ease the medical care. |
| 4. I was able to answer the question of the qualifications of the medical physician. |
| 5. I can answer the standing question of the willingness of the medical treatment. |
| 6. I can answer questions such as the willingness to ease medical care. |
| 7. I can answer the question of the removal of the medical will of The Ease of Health. |
| 8. I can answer legal questions that violate the soothing ordinance. |
| 9. I was able to answer the question of document preservation for Aon Medical. |
| 10. I was able to de-resolve the conflict of wishes of the relatives of the terminally ill. |
| 11. I was able to overcome the emotional stress of caring for terminally ill patients. |
| 12. I was able to overcome the sadness of facing the patient's death. |
| 13. I was able to overcome the grief of facing the death of my loved ones |
| 14. I was able to calmly face the physical problems of terminally ill patients. |
| 15. I was able to apply what I had learned to my family. |
